# Renalase: A Multi-Functional Signaling Molecule with Roles in Gastrointestinal Disease

**DOI:** 10.3390/cells10082006

**Published:** 2021-08-06

**Authors:** Thomas C. Pointer, Fred S. Gorelick, Gary V. Desir

**Affiliations:** 1 Department of Medicine, Yale School of Medicine, 333 Cedar St., New Haven, CT 06510, USA; Thomas.Pointer@Yale.Edu (T.C.P.); Fred.Gorelick@Yale.Edu (F.S.G.); 2 VA Connecticut Health Care System, 950 Campbell Avenue, West Haven, CT 06516, USA

**Keywords:** cell survival, renalase, PMCA4b, signaling, protein kinase, inflammation, pancreatitis, pancreatic cancer

## Abstract

The survival factor renalase (RNLS) is a recently discovered secretory protein with potent prosurvival and anti-inflammatory effects. Several evolutionarily conserved RNLS domains are critical to its function. These include a 20 aa site that encodes for its prosurvival effects. Its prosurvival effects are shown in GI disease models including acute cerulein pancreatitis. In rodent models of pancreatic cancer and human cancer tissues, increased RNLS expression promotes cancer cell survival but shortens life expectancy. This 37 kD protein can regulate cell signaling as an extracellular molecule and probably also at intracellular sites. Extracellular RNLS signals through a specific plasma membrane calcium export transporter; this interaction appears most relevant to acute injury and cancer. Preliminary studies using RNLS agonists and antagonists, as well as various preclinical disease models, suggest that the immunologic and prosurvival effects of RNLS will be relevant to diverse pathologies that include acute organ injuries and select cancers. Future studies should define the roles of RNLS in intestinal diseases, characterizing the RNLS-activated pathways linked to cell survival and developing therapeutic agents that can increase or decrease RNLS in relevant clinical settings.

## 1. Introduction

Renalase (RNLS) was first identified through the Mammalian Gene Collection Project [1]. Early work on RNLS focused on the proximal renal tubule of the kidney as a major source of circulating RNLS. Its tissue distribution and its enzymatic activity led to the name renalase. The reduced plasma renalase levels in chronic kidney disease (CKD) led to the posit that it might mediate complications of this condition. Its potential enzymatic functions, specifically its effects on catecholamine regulation and blood pressure, provided potential links between RNLS and its reduced levels with renal insufficiency, as well as CRF complications [1,2,3,4]. Subsequent studies have shown how RNLS can modulate the severity of acute injuries in experimental disease models that include the pancreas, liver, kidney, and heart. RNLS plasma and tissue levels were also reported to be dynamically regulated by environmental changes and injury. Given its prosurvival effects in acute injury settings, it is not surprising that RNLS was a molecular driver for select cancers. In preclinical studies, increasing extracellular RNLS levels has been shown to reduce a range of acute injuries while reducing RNLS levels has been shown to slow cancer growth. Recent studies find that extracellular RNLS signals through a specific plasma-membrane protein and elicits distinct intracellular signals. Although RNLS signaling is known in some systems, how the signals link to biologic responses remains unclear. Here, we review some of the foundational preclinical studies on this protein and then focus on its interesting structural and evolutionary features as well as its regulation and mechanisms of action.

## 2. Renalase Has Potential Roles in Disease: Findings from Preclinical Studies

### 2.1. Effects on Acute Injury

Initial studies of RNLS function examined whether it could reduce the effects of CRF using a murine model of acute kidney injury (AKI) that was treated with exogenous recombinant RNLS (rRNLS) [5]. The rRNLS treatment reduced plasma catecholamine levels and dramatically decreased ischemic renal injury, including inflammation. rRNLS was also protective in cellular models of oxidative injury and an in vivo murine model of acute ischemic renal injury. The protective effects of rRNLS we observed in the kidney prompted our examination of RNLS in another preclinical model of acute injury, acute pancreatitis.

Murine models of acute pancreatitis, using both isolated cells and in vivo preparations and treatments with cholecystokinin orthologue, cerulein, are often used for preclinical studies [6]. In the in vivo cerulein model, injury first occurs in the acinar cell, and later phases are mediated by inflammation. In both isolated acinar cells and in vivo, rRNLS reduced cerulein-induced injury [7]. Notably, the benefits of rRNLS in vivo were observed when administered well after the induction of pancreatitis, suggesting that RNLS can reduce inflammatory responses independent of its effects on the acinar cell in this model. The effects on inflammation are likely mediated in part by RNLS’s suppression of macrophage-dependent IL6 release, a pathway important to many forms of acute cellular injury. An unexpected finding in the pancreatitis study was that the plasma levels of renalase were dramatically reduced early in the course of acute pancreatitis but rebounded far above basal levels during recovery. This suggests that plasma RNLS levels are dynamically regulated in both the acute and recovery phases of injury.

Subsequent studies by others have suggested that RNLS may have a role in mediating other forms of gastrointestinal injury. RNLS was shown to reduce oxidative liver injury in cellular and an in vivo murine model of ischemia/reperfusion injury that was superimposed on models of fatty liver disease [8]. Similar to acute pancreatitis effects, exogenous rRNLS reduced injury in intact liver and related cellular disease models. Although its protective mechanism is not fully understood, RNLS reduced mitochondrial injury in liver cells. A high fat diet reduced both hepatic RNLS gene expression and plasma RNLS levels, underscoring the potential importance of environmental regulation of tissue RNLS expression. It also suggests that plasma levels may be relevant to acute injury responses. Environmental oxidative stress changed RNLS expression in small intestinal crypts and cultured transformed intestinal cells [9]. Similar to the liver, increasing RNLS levels (by transfection) reduced oxidative injury in cultured intestinal cells. Together, these studies suggest that environmental factors could modulate tissue and plasma levels of RNLS. It also suggests that RNLS could have essential roles in modulating injury in the pancreas, liver, and small intestine. Together, these studies underscore the potential for using RNLS agonists as therapeutic agents to modulate the acute injury severity.

### 2.2. Effects on Cancer

The prominent prosurvival effects of RNLS on multiple cell types raised the possibility that it might have a similar role in cancer. RNLS mRNA levels were increased in PDAC and melanoma and other cancers. Our first study examined a potential role for RNLS in pancreatic cancer (PDAC) using human tissues, cultured cells, and a murine model [10]. Tissues levels of RNLS in PDAC corresponded to patient survival inversely; the higher RNLS levels the shorter the survival. Inhibition of RNLS using siRNA or an inhibitory monoclonal antibody to RNLS reduced PDAC growth in cultured cells and in vivo. Similar studies in patients with melanoma showed that RNLS tissue levels are inversely related to survival and required for experimental melanoma growth [11]. Using immune-localization to determine the identity of cancer cells associated with RNLS, researchers found that much of the RNLS immunoreactivity was in tumor-associated macrophages as well as melanoma cells. The localization of RNLS in cancer and immune cells suggests that more than one cell type might mediate the effects of RNLS on cancer cell growth. Together, these promising findings suggest that agents that inhibit the effects of RNLS in select cancers could be attractive new therapeutic tools and should also encourage investigations of RNLS in other cancer types.

Collectively, these models of acute injury and cancer show prosurvival roles for RNLS. Depending on the disease context, RNLS could either be of benefit (in acute injury) or detriment (in cancer) to the organism.

### 2.3. The Cell Growth and Anti-Inflammatory Activities of RNLS Localize to a Specific Site

The biologic activities have been examined by using sequence homology analysis and studies specific regions of the RNLS protein. Most relevant to this review, its cell growth and anti-inflammatory activities are found in a 20 amino acid peptide (aa 220 to 239 of human RNLS), which is referred to as the RP-220 peptide [12]. The critical role of the RP-220 is underscored by the fact that it has been used to identity the cellular RNLS receptor (Section 6.2.1) and also as the target for generating a humanized monoclonal antibody (m28) that greatly reduces pancreatic cancer and melanoma growth in cells and in vivo (Guo and Hollander).

Together, these studies provided compelling evidence of key regulatory roles for RNLS as a prosurvival factor and modulatory of inflammatory responses in preclinical models of acute tissue injury as well as in cancer. To understand RNLS’s mechanism and as a critical step in developing agents that could modify or mimic its biologic activities, we have begun to study its structure and complex effects on cells including its links to distinct intracellular signals. This will be the subject of the remainder of this review.

## 3. RNLS Forms, Conservation, Structure, and Expression

### 3.1. RNLS Isoforms

**Figure 1:**
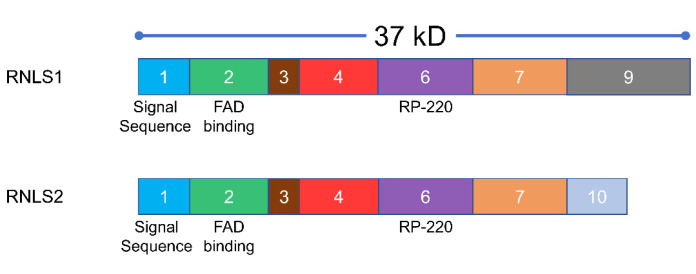
Renalase domains are conserved between RNLS1 and RNLS2, which only differ in the C-terminal site because of differences in splicing. The RP-220 site has prosurvival activity; it is also target for an inhibitory RNLS antibody.

The human RNLS (hRNLS) gene contains approximately 300,000 nucleotides across 11 exons and is located on chromosome 10 [13]. Two major isoforms, RNLS1 and RNLS2, are found in humans (Figure 1). RNLS1 has 1477 nucleotides, 7 exons, 342 residues, and a theoretical molecular mass of 37.85 kD. RNLS2 has 2107 nucleotides, 5 exons, 315 residues, and a theoretical molecular mass of 34.95 kD [14]. Potential distinct splice variants were also identified in humans [13,15,16]. The other variants, including much the much shorter RNLS 3 and 4 (RNLS3 and RNLS4), would lack oxidase function if expressed [12]. The potential existence of these variants complicates the characterization of RNLS-dependent responses. Further, RNLS1 antibodies have been shown to recognize RNLS2, suggesting that effects previously attributed to RNLS1 could be RNLS2 or due to a combination of the isoforms [14].

In contrast to the two RNLS isoforms expressed in humans, only one form is found in murine tissues; the murine form is most similar to human RNLS1 (hRNLS). hRNLS1 and hRNLS2 only differ in their C-terminal domains, and the key functional domains are conserved between the isoforms (Figure 1). This includes N-terminal signal sequences and a FAD-binding domain and the RP-220 site in exon 6 (see Section 3.3). The enzymatic NAD oxidase of RNLS is in the N-terminal region and a putative catecholamine-binding motif is found in the center of the molecule. Details of RNLS structure are discussed below (see Section 3.3).

### 3.2. RNLS Sequence Conservation

Soon after the discovery of RNLS in 2005, investigations into the evolutionary history of the protein yielded surprising findings. One study of cyanobacteria-originated metazoan/fungi proteins (COPs) developed a hypothesis that RNLS originated from nuclear-localized plastid-like DNA (nupDNA) fragments from cyanobacteria [17]. This study places the horizontal gene transfer event that included RNLS around 700 million years ago between the first eukaryote naissance (1200 million years ago) and the first metazoan appearance (570 million years ago) [18,19]. After this transfer, the gene would have diverged to produce the lineages and genes that we see today with an accumulation of changes. The conservation of RNLS sequence domains is supported by more recent work that found overall sequence similarity between plant gene products and mammalian RNLS [13]. Through evolution, it is likely that the function of this protein has evolved and may be reflected in changes in sequence. However, the existence of the protein dating so far back in the tree of life suggests integral, but potentially changing, function(s).

We have examined segments of the RNLS sequence that encode for predicted or known activities to provide insights into their relative functional importance [20]. We included alignment of the hRNLS amino acid sequence to RNLS genes found in *Pan troglodytes* (chimpanzee) and *Rattus norvegicus* (rat). The RNLS N-terminus is thought to be necessary for FAD-binding and some of the oxidase activity of RNLS [21,22]. Of the first 15 amino acids, 11 are identical and 3 more have been substituted by conserved residues. The RP-220 region that has effects on cell survival and inflammation (discussed below) is also highly conserved. Of the 20 residues in the region (aa 220-239 of hRNLS 1 and 2), 16 are identical, and 1 has been substituted by a residue with similar properties among these three species.

We have also analyzed sequences from non-vertebrates, such as *Actinia tenebrosa* (Australian red waratah sea anemone). In the 15 residues that aligned with the sequence of the N-terminal of hRNLS, 9 are identical and 4 have been substituted by residues with similar properties. The identical residues include the GXGXXG motif of a Rossman-fold, a necessary component for the binding of FAD to the protein [21,23]. For the RP-220 region, nine residues are identical and five more have been substituted by residues with conserved properties. When comparing the sequences, research found that other regions show high conservation. For example, the residues 190–200 are identical throughout all four sequences, suggesting that it has an important but yet to be identified biological function. This site is also predicted to be exposed on the surface of RNLS in a potential binding region [24].

### 3.3. RNLS Structure

The three-dimensional RNLS1 crystal structure has been determined using purified recombinant soluble human renalase expressed in E. coli bacteria [24,25]. A prominent feature of the structure showed a FAD-binding domain (Rossmann fold) [24]. This three-dimensional structure is similar to MAO-A, MAO-B, and polyamine oxidase, but lacks some of the residues predicted to be essential for amine oxidation catalysis [15,26,27]. Of note, analysis of RNLS1’s crystal structure revealed no disulfide bonds. This was unexpected since the protein contains 12 cysteine residues [15]. Unless specifically engineered to do so, bacteria lack the machinery needed to properly align disulfide residues. This makes recombinant proteins made in bacteria susceptible to misfolding and could produce misleading results relating to protein structure and function.

The potential of RNLS to metabolize amines was identified as a possible function based on its sequence homology with other flavoproteins. More recently, RNLS was noted to lack the helical domain found in other flavin-dependent amine oxidases (FAO) superfamily members. In other FAO proteins, this helical domain appears to be important in substrate recognition/binding and membrane interactions [28]. Interestingly, the RNLS NAD(P)H oxidase activity increased after mutations that inserted more positively charged residues in the active site [29]. The association with FAD with RNLS is thought to be non-covalent [21]. It has since been shown that the large hydrophilic cavity of RNLS seems appropriate for a charged nicotinamide substrate [28]. The RNLS cleft that associates with nicotinamide dinucleotides does not appear to exhibit hydrogen bonds with ribose; instead, it interacts with the pyrophosphate moiety [30]. Whether RNLS binds PPi and the potential role of this as a signaling moiety in such processes as glucose metabolism deserve further consideration [31]. Overall, the structure of RNLS1 has been well characterized, especially those regions involved in FAD and NAD(P)H binding; the importance of RNLS interactions with FAD and NAD are discussed below (see Section 4). Other three-dimensional regions (including RP-220 that contains important biologic activities) are not as well understood and deserve further investigation, perhaps using recombinant proteins engineered to ensure proper folding.

## 4. RNLS Cell Biology

### 4.1. Cellular Distribution/Secretion

RNLS has an n-terminal signal sequence that appears to be cleaved in secreted forms [1,16,32,33,34,35,36]. RNLS1 and RNLS2 have been shown to contain the FAD-binding moiety and N-terminal peptide before secretion. One group detected RNLS in the urine and found that it lacked this N-terminal signal peptide [21]. The FAD-binding region forms an alpha helix and a beta-strand critical to the formation of the Rossman fold, according to in silico studies [21]. Those authors suggest that this truncated RNLS is incapable of binding FAD, meaning any effects of this truncated RNLS are FAD-independent [22]. It may be relevant that our unpublished data suggest that RNLS might be secreted, at least in part, through a non-canonical secretory pathway and may also retain its signal peptide that does not exhibit truncation of the putative FAD binding domain. Additional studies are needed to define the mechanism(s) of RNLS secretion, how secretion is stimulated, and the processing of its signal peptide. The intracellular itinerary of RNLS may vary in an isoform and tissue-dependent manner and be affected by local and systemic environmental factors.

The intracellular distribution of RNLS has not been fully defined; in some cells, it has been localized to distinct intracellular structures and in others at or near the plasma membrane.

### 4.2. RNLS Cellular Expression Is Regulated

**Figure 2:**
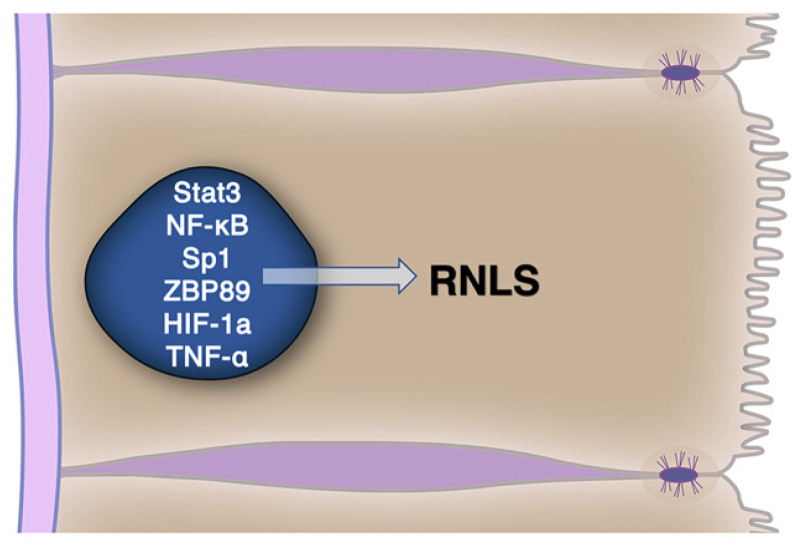
Renalase gene expression is regulated by a range of transcription factors, several of which are linked to proinflammatory responses.

Tissue levels of RNLS can be regulated and this response is likely to modulate its biologic effects and as well as its roles in diseases involving acute injury and in cancer. Several factors (Figure 2) have been shown to upregulate RNLS gene expression including STAT3, Sp1, ZBP89, NF-κB, HIF-1α, and TNF-α in various tissues [9,37,38]. For STAT3, a positive feedback loop with RNLS has been suggested with STAT3 enhancing RNLS production and RNLS, in turn, increasing STAT3 activation [11,37].

To investigate these pathways as well as RNLS function, our laboratory generated a rabbit monoclonal antibody (m28) to the human RNLS aa 220-239 RP-220 peptide that encodes for its prosurvival activity. The antibody inhibited the growth of cultured pancreatic cancer cells as well as in vivo tumors growth in a murine model of pancreatic cancer. This was associated with a significant reduction in both RNLS expression and STAT3 (both phosphorylated and total), also supporting the presence of a positive feedback loop. The inhibition of RNLS and reduced STAT3 signaling were associated with decreased cell proliferation and increased apoptosis [11]. Similar RNLS-dependent STAT3 signaling was found in Panc1 pancreatic cancer cells and the human kidney HK-2 cells [10]. A link between RNLS expression and STAT3 was also observed in a model of fatty liver disease [8]. However, a RNLS–STAT3 interaction was not found in macrophages, suggesting that the relationship could vary among tissues [11].

Another potentially important RNLS regulatory pathway that may be relevant in the context of both ischemic injury and cancer relates to the four potential HIF-1α-binding motifs identified within the promoter region of RNLS. These are responsible for HIF-1α-induced increases in RNLS expression. In HIF-1 α knockdown mice, the genetic deletion worsened experimental ischemia/reperfusion (I/R) cardiac injury compared to wild-type (WT) mice. Injection of RNLS reversed the effects of HIF-1α deletion on the excess deterioration of cardiac function [39]. This indicates a significant role for RNLS in mediating the protective effects of HIF-1 α, at least in the context of I/R cardiac injury. Together, the involvement of these factors suggests complex, multi-faceted roles for RNLS action and regulation of its levels that will likely affect divergent and distinct signaling pathways.

## 5. Renalase Signaling

### 5.1. Enzymatic Activities of RNLS

The potential enzymatic functions of RNLS1 were the focus of the original studies of the protein [1,4,26,27]. Although RNLS’s in catecholamine metabolism remains unclear (although direct or indirect effects are very likely), interests in the enzymatic properties of RNLS, which may be most relevant to the function of an intracellular pool, has shifted to the oxidative metabolism of NAD(P)H [30,40,41]. Though the scope of studies has since broadened to include other functions of the protein (specifically its signaling capabilities), it is still critical to consider the enzymatic properties when determining the role of RNLS1 in disease and recovery.

#### 5.1.1. Enzymatic Activities and NAD

Our laboratory has predicted and shown evidence that RNLS functions as a NADH oxidase [42,43,44]. Other studies showed that RNLS oxidizes and epimerizes α-NAD(P)H molecules [24,45,46]. Subsequent studies showed that this oxidation takes place at the 2- or 6-position of the nicotinamide ring, rather than the metabolically active 4-position. This supports a role as a “scavenging” enzyme that prevents the buildup of unusable substrates [41]. That RNLS can convert metabolically inert 2 or 6 NADH to active 4-NAD+ may have biologic relevance since a reduction of NAD/NADH is observed in the myocardium of RNLS knockout mice [47]. NAD metabolism is tightly coupled to cellular energy metabolism [48]. That cellular ATP levels are also dramatically reduced in RNLS knockout mice (unpublished data) suggests that RNLS, particularly an intracellular pool that modulates NAD metabolism, could have profound effects on cellular energy production. Overall, this function is compatible with the larger theme of RNLS as a prosurvival factor, and this functionality might apply to both its intracellular and extracellular functions.

#### 5.1.2. Other Potential Enzymatic Activities

Findings related to the potential catecholamine-metabolizing activity of RNLS vary widely [1,3,13,25,42,46,49]. One study claimed that although RNLS might bind epinephrine, this binding is distant from the oxidase site and does not affect NADPH binding or the flavin environment [46]. Whether RNLS directly regulates catecholamine levels enzymatically remains unclear. However, it does appear to have a prominent effect on plasma catecholamine levels with plasma epinephrine levels dramatically increasing in RNLS knockout mice [16,47]. In the context of its enzymatic activities, the full-length recombinant human RNLS (rhRNLS) used for published assays have used proteins made in yeast that may not have been properly folded and could provide misleading information relating to its enzymatic activities. In addition, RNLS may interact with other molecules that either modify its activities or serve as biologic targets. These functions of RNLS have likely to have changed significantly over time and across lineages. Some functions may be remnants of ancient forms of the protein that are now redundant with other proteins.

## 6. Signaling by Extracellular RNLS

### 6.1. The Growth and Modulatory Effects of RNLS Are Found in a Specific RNLS Site

As mentioned above, we have used models of acute renal injury and truncated rRNLS constructs, to identify the prosurvival and anti-inflammatory effects of RNLS and localized the activity to a 20 amino acid peptide (aa 220 to 239 of human RNLS referred to as RP-220) [12]. The site is located far from the region of the molecule that contains enzymatic activity. The Rp-220 peptide replicates many of the biologic effects and signaling pathways activated by full-length RNLS [50,51]. It also has one of the most highly conserved amino acid sequences through evolution in the molecule (discussed elsewhere). The importance of the RP-220 sequence to the function of RNLS is underscored by the finding that monoclonal antibodies to the RP-220 peptide can be potent inhibitors of cancer growth [10,11].

### 6.2. Extracellular RNLS Stimulates Intracellular Signaling

Although the enzymatic function of RNLS was the initial focus of our laboratory, in later structure–function studies, we discovered that RNLS also has independent functions as an extracellular signaling factor that plays an important role in modulating inflammation and recovery from acute injury [7]. We find that many of RNLS’s prosurvival functions are independent of the oxidase properties of RNLS [12].

#### 6.2.1. Identification of a Plasma Membrane Receptor for RNLS

Since treatments adding rRNLS or RP-220 to the extracellular compartment resulted in biologic responses, we anticipated that the molecules were likely acted by binding to a protein on the cell surface. To identify the protein, we covalently linked the RP-220 peptide to membrane proteins. The bound proteins identified by mass spectroscopy included plasma-membrane calcium export pump, PMCA4b (plasma-membrane calcium ATPase 4b), as a leading candidate. Its interaction with RNLS is also shown by co-immunoprecipitation. PMCA4b is concentrated on the basolateral membrane of acinar cells, suggesting that it responds to extracellular RNLS. This was confirmed by functional studies that included showing that PMCA4b downregulation by siRNA or its inhibition by caloxin 1b1 blocked the prosurvival effects of RNLS [7,50]. Since PMCA4b is a widely distributed plasma-membrane transporter, it may mediate the effects of extracellular RNLS in other tissues. Further details relating to PMCA4b signaling are provided in Section 6.2.2.

Limited studies suggest that RNLS and PMCA4b may interact with other cell surface receptors, such as the G-protein-sensitive estrogen receptor (GPER), which can downregulate PMCA4b when activated with estradiol (Section 6.2.2). Likely, the signals elicited by extracellular RNLS described in the following section can be modified by other yet unknown signaling mechanisms.

#### 6.2.2. RNLS Stimulates Distinct Intracellular Signals

**Figure 3:**
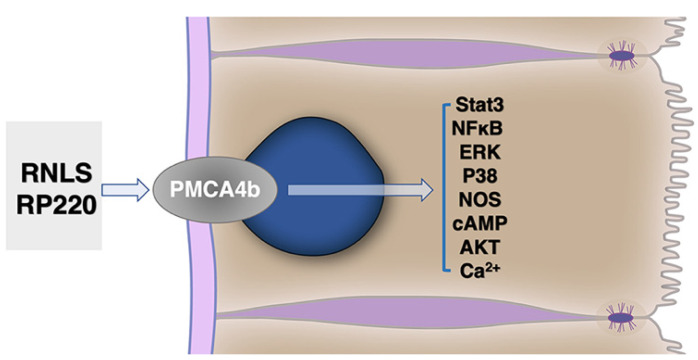
Renalase signaling through the plasma-membrane calcium ATPase (PMCA4b) is linked to the stimulation of multiple downstream signals.

We have examined the effects of extracellular RNLS on intracellular-regulated protein kinase pathways. These include activation of AKT, ERK, p38, B cell lymphoma 2, and inhibition of c-Jun K-terminal kinase (Figure 3) [12]. The RP-220 peptide sequence and related peptides common to all known forms of RNLS have been used in most of our studies. The RP-220 site (RNLS1 aa 220-239) has no known enzymatic activities, but it has demonstrated a prosurvival effect after acute injury to kidney, heart, and corneal epithelial cells [12,51,52]. In select systems, the signals induced by full-length RNLS have been replicated using RP-220 [12,50,52]. The retained functionality when oxidase- and FAD-independent activities are absent provides compelling evidence of the signaling function of this RNLS site.

Some of the same signaling pathways activated by RNLS and RP-220 have also been linked to PMCA4b signaling. These include the Ras/Raf/MEK/ERK, p38, NOS, NF-κB, cAMP, and AKT pathways [12,53,54,55]. One study described an interesting potential feedback loop, finding that p38 (which is upregulated with RNLS treatment) induces degradation and downregulation of PMCA4b [55]. When treated with a p38 inhibitor, PMCA4b degradation was blocked and calcium clearance from the cell increased. This p38-dependent response could be therapeutically important in certain types of injury, such as acute pancreatitis, where calcium efflux is a key modulator of injury and recovery [56]. It has also been suggested that inhibition of PMCA4b could increase NO accumulation [57]. This suggests the possibility that if RNLS activates PMCA4b, NO accumulation could be reduced, leading to less severe injury or more rapid recovery. NF-κB-regulated RNLS expression has also been shown to cause increased phosphorylation of AKT through a PMCA4b-dependent mechanism [58]. In B16-F10 cells, PMCA4b was also found to have a key role in RNLS-dependent ERK and STAT3 phosphorylation [11]. Together, these studies demonstrate that RNLS can affect several distinct cell signaling pathways in a PMCA4b-dependent manner.

PMCA4b has a PDZ domain, a protein motif that often links transmembrane proteins or proteins that concentrate near membranes. In the case of PMCA4b, it can link the calcium exporter to other signaling pathways [59]. For example, PDZ-dependent signaling of PMCA4 was found to be a negative regulator of NOS-I [60]. PDZ domains interact with other proteins that often have scaffolding functions and that can anchor multiple protein signaling cassettes and do so in a tissue-dependent manner [61,62,63]. The interactions of the PDZ domain have been found in a variety of cell types including neuronal cells, smooth muscle cells, and cardiomyocytes [60,61,64,65]. This model is potentially crucial to understanding the role of PMCA4b and the PDZ domain and might provide one mechanism to relate cellular Ca^2+^ levels to NO production and other cells signals [53] More recently, it was determined that constitutive ERK activation is PDZ-dependent, while activation by a specific agonist, G-1, is PDZ-independent [66]. This variety in signaling could help promote signal specificity and have distinct outcomes in a tissue-specific manner.

The PMCA4b PDZ domain appears to enable the membrane protein GPER (G-protein estrogen receptor) to be coupled in a regulatory manner to PMCA4b through the linker protein, PSD95 (post-synaptic density 95). Activation of GPER by estradiol results in the tyrosine phosphorylation of PMCA4b and downregulation of its calcium-export activity [57,67]. This observation establishes an important precedent for PMCA4b, and hence RNLS-signaling, to be modified by another tightly coupled receptor. In the case of GPER, its activation could in theory counteract the effects of RNLS and reduce the prosurvival and anti-inflammatory effects of RNLS. It will be important to consider how PMCA4b activity might be modulated by PDZ-linked proteins as well as how PMCA4b itself might change the function of linked proteins.

## 7. RNLS Presence in Human Intestine

**Figure 4:**
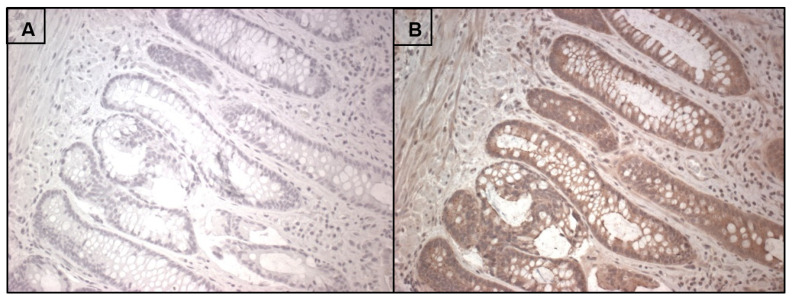
Renalase immunoreactivity in normal human colonic mucosa labeled with anti-m28-RNLS or 2.5% horse serum using an immunoperoxidase method labeled with (**A**) no primary antibody as a negative control; (**B**) anti-renalase m28 antibody. The specific brown RNLS labeling (**B**) is most prominent on the epithelial cells lining the crypts of the intestinal glands. Tissue samples were de-identified and acquired through a Yale HIC-approved protocol by Dr. Marie Robert.

Though discovered and primarily studied in the kidney, RNLS gene expression can be found in virtually every organ including those of the gastrointestinal tract. Our preliminary studies have also detected RNLS in select gastrointestinal tissues using immunocytochemistry. For example, we used the m28 antibody to detect immunoreactivity (protocol Appendix A) in colonic mucosal cells (Figure 4). This tissue shows RNLS labeling (dark brown stain not seen in the control lacking the primary antibody) can be seen in human colonic cells surface epithelial cells throughout the crypt and scattered throughout the submucosa. The presence of RNLS in cells at baseline suggests that it may have a role in tissue homeostasis. A model characterizing the role of RNLS in one or more of these tissues may apply to other tissues. Other proteins essential for RNLS signaling, such as the isoform 4b of plasma membrane calcium ATPase (PMCA4b), have also been identified in these tissues [68,69,70]. A model describing the role of RNLS in reducing injury and modulating inflammation across these systems would be of great importance. The potential role of RNLS as a modulator of inflammation may be best studied in the intestine due to its baseline pro-inflammatory state. Given the strong moderating effect of RNLS on the inflammatory and immune response, we speculate that RNLS could contribute to gut homeostasis by modulating the response to foreign molecules that activate innate immune responses.

## 8. Cell and Tissue-Specific RNLS Responses

### 8.1. Macrophages

There is growing evidence that immune cells are an important target for RNLS signaling [51,71,72]. Macrophages are often referred to as having characteristics of an M1 (proinflammatory) or M2 (anti-inflammatory) phenotype. Exposure of macrophages to RNLS has been shown to increase RNLS production and to drive M2 phenotype [11]. It has also been suggested that dysregulated RNLS signaling could influence macrophages toward a tumor-promoting, M2-like phenotype [51]. Exposure of activated (M1) macrophages to RNLS has been shown to block inflammasome activation and IL1β production [51]. Although macrophages have been shown to express PMCA4 [73], whether RNLS signals through this transporter remains to be explored.

### 8.2. Others

In a study in the liver, a marker of mature macrophages, Adgre1, was found to be significantly higher in RNLS KO mice during the progression of nonalcoholic steatohepatitis (NASH). It is suggested that this hepatocyte dysfunction and hepatic fibrosis progression could be connected to the absence of RNLS. Additionally, the absence of RNLS may cause an increase in oxidative stress and bring on macrophage infiltration in these tissues [58].

### 8.3. Cancer

As discussed in Section 2.2, RNLS1 promotes cell survival in most cancer cells as shown for pancreatic cancer and melanoma. In PDAC cells, siRNA and anti-RNLS1 antibodies decreased the survival of PDAC cells [10]. The correlation between cancer and RNLS1 levels in the kidney has led some to suggest that tissue RNLS1 could be a biomarker for renal tumors [74]. Elevated RNLS1 levels are also found to be associated with breast cancer [75]. A systematic evaluation of RNLS in gastrointestinal and non-GI tumors is needed.

## 9. Conclusions/Future Studies

It has been established that RNLS has a potent prosurvival role in several different systems. The distinct enzymatic and signaling properties of RNLS have potentially profound effects on injury and recovery. Since RNLS is abundantly found in many non-diseased tissues, it seems certain that it will also have a role in tissue homeostasis. That tissue RNLS levels can be modified by environmental factors such as a high-fat diet suggests such changes could modify injury responses. The RNLS properties that modulate acute injury responses likely have key roles in the malignant behavior of some cancers. Although direct effects of RNLS on parenchymal cells (e.g., pancreatic acinar cells) and cancer cells (pancreatic and melanoma) have been observed, it is likely that RNLS acts on other cell types that modulate disease responses. Thus, though the immunologic effects of RNLS are still being described, they likely have a role in modulating injury responses and establishing and maintaining tumor niches.

Our studies of RNLS as a potential therapeutic tool for use in the treatment of acute injury as well in select cancers have focused on the extracellular RNLS pool. Delivery of intact RNLS has been shown to decrease the severity of a range of acute injuries. RNLS inhibition using a specific monoclonal antibody has been shown to reduce the growth of select cancers. Although these findings represent attractive avenues to therapy, a more refined understanding of RNLS function may allow for the creation of more effective and specific therapeutic tools. This includes understanding details of the RNLS signaling pathways and structure–function relationships of the molecule.

In closing, RNLS is a multi-functional protein with activities that are broadly important for maintaining tissue homeostasis and for resolving acute tissue injury. It is also a driver for select cancers. Developing agents that are RNLS agonists or antagonists might have broad therapeutic applications.

## Data Availability

Not applicable.

## Appendix A

### Appendix A.1. Tissue Specimens

De-identified human samples of normal stomach, duodenum, jejunum, ileum, and colon, as well as a pancreatic cancer specimen, were obtained from Yale Surgical Pathology and approved for exemption by the VA Connecticut Healthcare Human Investigation Committee.

### Appendix A.2. Immunohistochemistry

Specimens (5 µm) were embedded in paraffin and attached to glass slides. The sections were deparaffinized and hydrated followed by antigen retrieval by steaming and a citrate buffer for 20 m. Samples were then blocked in DAKO Dual endogenous enzyme block (Agilent, Santa Clara, CA, USA) for 10 min followed by blocking in normal horse serum, 2.5% (Vector Laboratories, Burlingame, CA, USA) for 1 h before incubation with m28 anti-RNLS (1:500 dilution) in TBS/1% Tween20 overnight, or 2.5% normal horse serum overnight. ImmPRESS peroxidase-anti-rabbit IgG (Vector Laboratories, Burlingame, CA, USA) detected the primary antibodies. A Vector DAB substrate kit was used to develop the brown color. The slides were finally stained with hematoxylin (Sigma Aldrich, St. Louis, MO, USA) and rehydrated. Coverslips were fixed to the slides using Vectamount (Vector Laboratories) and observed via light microscopy.
